# *Treponema* spp. Isolated from Bovine Digital Dermatitis Display Different Pathogenicity in a Murine Abscess Model

**DOI:** 10.3390/microorganisms8101507

**Published:** 2020-09-30

**Authors:** Rakel Arrazuria, Cameron G. Knight, Priyoshi Lahiri, Eduardo R. Cobo, Herman W. Barkema, Jeroen De Buck

**Affiliations:** 1 Department of Production Animal Health, Faculty of Veterinary Medicine, University of Calgary, Calgary, AB T2N1N4, Canada; vetrakel@gmail.com (R.A.); priyoshi.lahiri@ucalgary.ca (P.L.); ecobo@ucalgary.ca (E.R.C.); barkema@ucalgary.ca (H.W.B.); 2 Department of Veterinary Clinical and Diagnostic Sciences, Veterinary Medicine, University of Calgary, Calgary, AB T2N1N4, Canada; cgknight@ucalgary.ca

**Keywords:** *Treponema phagedenis*, *Treponema pedis*, *Treponema medium*, digital dermatitis, mouse abscess model

## Abstract

Digital dermatitis (DD) causes lameness in cattle with substantial negative impact on sustainability and animal welfare. Although several species of *Treponema* bacteria have been isolated from various DD stages, their individual or synergistic roles in the initiation or development of lesions remain largely unknown. The objective of this study was to compare effects of the three most common *Treponema* species isolated from DD lesions in cattle (*T. phagedenis, T. medium and T. pedis*), both as individual and as mixed inoculations, in a murine abscess model. A total of 10^9^ or 5 × 10^8^ *Treponema* spp. were inoculated subcutaneously, and produced abscess was studied after 7 days post infection. There were no synergistic effects when two or three species were inoculated together; however, *T. medium* produced the largest abscesses, whereas those produced by *T. phagedenis* were the smallest and least severe. *Treponema* species were cultured from skin lesions at 7 days post infection and, additionally, from the kidneys of some mice (2/5), confirming systemic infection may occur. Taken together, these findings suggest that *T. medium* and *T. pedis* may have more important roles in DD lesion initiation and development than *T. phagedenis*.

## 1. Introduction

Digital dermatitis (DD) is a foot disease that causes lameness in cattle. It is characterized by an inflammatory dermatitis of the digital skin, most commonly on the plantar aspect of the interdigital cleft [1]. At the outset, DD is an ulcerative epidermal lesion and often progresses to a painful, proliferative, nodular mass in the interdigital space just proximal to the heel bulb. These lesions generally do not heal spontaneously and may become severe, resulting in loss of the hoof [2]. On many dairy farms, DD is endemic and causes substantial economic loss by reducing both milk yield [3] and reproductive performance [4], and also affecting animal welfare [5].

Despite many studies focused on elucidating causative agents of DD, the exact etiology is uncertain. In cattle with DD, lesions typically contain a variety of bacteria, including *Treponema, Fusobacterium, Bacteroides, Campylobacter, Mycoplasma* and *Porphyromonas* [6,7,8]. However, treponemes are the only bacteria with substantial evidence of an etiological association [9]. Although various *Treponema* spp. are consistently identified in active bovine DD lesions [8,10,11,12], the exact role of these spirochetes in the aetiopathogenesis of DD remains unclear [13]. *Treponema phagedenis*, *T. medium*, *T. pedis* are among the most commonly detected species in DD lesions [8,10,11,14,15]. Interestingly, DD-associated treponemes have also been associated with other hoof-related animal diseases, including contagious ovine digital dermatitis [16] and elk hoof disease [17]. In addition, they have also been detected in lesions of bovine ischaemic teat necrosis [18] and in skin and tail lesions of pigs [19].

Active DD lesions have a higher *Treponema* spp. diversity than either chronic or initial stages of the disease [8,11,14]. In addition, different species of *Treponema* are associated with different DD stages [14], suggesting their interactions could be related to disease progression. In other *Treponema*-associated diseases, such as periodontal disease in humans, the association of treponemes and other organisms extends beyond simple cohabitation and is associated with nutritional and pathogenic synergies [20]. For example, *Treponema denticola* and *Porphyromonas gingivalis* are strongly associated with chronic periodontitis where they exhibit metabolic symbioses [21,22], synergistic polymicrobial biofilm development [23,24] or synergistic virulence upon coinfection in animal models of disease [25,26]. In addition, the bacterial complex named “red-complex”, consisting of *P. gingivalis*, *T. denticola* and *Tannerella forsythia*, is strongly related to advanced periodontal lesions [27], pointing at complex polymicrobial synergies and dysbiosis as the cause of the disease [28].

Although various animal models of periodontitis have been developed [29], murine subcutaneous abscess models have been widely used to determine the virulence and pathogenicity of various periodontitis-associated bacteria, including several *Treponema* spp. [26,30,31]. When a murine subcutaneous abscess model was used to characterize pathogenicity of several *Treponema phagedenis* isolated from DD, there were differences among these strains in their ability to produce lesions in mice [32]. Despite many attempts to reproduce DD in both cattle and sheep [33,34,35], no well-characterized ruminant model of this disease, suitable for large-scale studies, has been reported. Therefore, in the present study, pathogenicity of DD-associated spirochetes was evaluated using a murine subcutaneous abscess model.

Specific roles of various *Treponema* spp. in DD skin lesion initiation or development are unknown. Our objective was to investigate the effects of the three most common *Treponema* spp. isolated from DD skin lesions (*T. phagedenis, T. medium and T. pedis*), both individually and as mixed inoculations, in a murine subcutaneous abscess model.

## 2. Materials and Methods

### 2.1. Experimental Design

C57BL/6J (6-week old) mice were purchased from Jackson Laboratories (Bar Harbor, ME, USA) and acclimated to the facility for 1 week prior to infection. As in previous studies using the murine subcutaneous abscess model [26,30,32], female mice were used. All experimental protocols involving animals were reviewed and approved by the University of Calgary Health Sciences Animal Care Committee (AC17-0201, 28-11-2017). 

**Figure 1:**
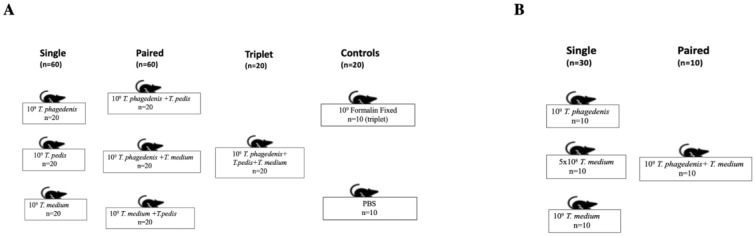
Experimental designs of subcutaneous inoculations. (**A**) Experiment carried out with single, paired and triplet *Treponema* spp. inoculum and two controls (formalin-fixed bacteria and PBS). (**B**) Experiment 2, carried out with single and paired *Treponema* spp. infection.

Two experiments were performed. In Experiment 1, mice were allocated into seven experimental groups (*n* = 20/group) and two control groups (*n* = 10/group) (Figure 1A). Three groups were challenged with an inoculum containing 1 × 10^9^ of a single *Treponema* species (*T. phagedenis, T. medium* and *T. pedis*). Another three groups were exposed to a combination of equal numbers of two *Treponema* species (total of 1 × 10^9^ bacteria of *T. phagedenis + T. medium; T. phagedenis + T. pedis* or *T. medium + T. pedis*), and the seventh group was challenged with a combination of three *Treponema* species (*T. phagedenis + T. medium + T. pedis*) at the same concentration (total of 1 × 10^9^). One of the control groups was challenged with a combination of three formalin-killed *Treponema* species (total of 1 × 10^9^) and the second control group was inoculated with phosphate buffered saline (PBS) (Figure 1A).

Experiment 2 was performed to test the reproducibility of some of the obtained results and to investigate dose effects on abscess size (Figure 1B). Mice were allocated into four groups (*n* = 10/group). Three groups were challenged with a single *Treponema* species at two concentrations (5 × 10^8^ or 1 × 10^9^), whereas the fourth group was inoculated with a mixture of equal numbers of *T. phagedenis* and *T. medium* (total of 1 × 10^9^).

### 2.2. Treponema spp. Inoculum

*Treponema* spp. isolated from bovine DD cases were used to inoculate mice. Glycerol stocks were grown at 37 °C for 7 days in anaerobic novel oral spirochete media (NOS) containing 10% bovine fetal serum and 10% rabbit serum, supplemented with rifampicin and enrofloxacin at 5 μg/mL, as previously described [36]. All cultures were incubated in an anaerobic chamber (85% N_2_, 5% CO_2_ and 10% H_2_). Bacterial concentration, active motility and the absence of spherical bodies were determined by counting spiral forms under a dark-field microscope, using a Petroff-Hausser chamber. For inoculum preparation, cultures were washed with pre-reduced PBS, and final concentrations of 1 × 10^9^ or 2 × 10^9^ spirochetes/mL were obtained. The inoculum (always 500 μL) was injected subcutaneously along the dorsal midline, between the scapulae. To ensure cell viability, all inoculations were performed within 15 min after inoculum preparation.

### 2.3. Necropsy

Mice were euthanized 7 days after inoculation. The site of injection into the dorsal skin was photographed and a rectangle of dorsolateral skin that included the injection site was removed, using blunt dissection to separate the subcutis from underlying muscle and fascia. The excised skin was inverted and the deep surface of the injection site photographed. Height, width and depth of each abscess were measured with a caliper and its volume calculated (mm^3^). Lesions from 50% of the mice of each group (*n* = 10) were processed for histopathology. To that end, the skin was pinned flat to cardboard to prevent curling and immersed in 10% formalin until fully fixed. Each lesion from the other 50% of the mice in each group (*n* = 10) was sectioned with a sterile scalpel blade into three parts; these were processed for bacterial culture, *Treponema* spp. qPCR and transcriptional gene expression of GAPDH, CXCL-1, IL-10 and TGF-β (PPM02991B). To detect potential systemic dissemination of *Treponema* spp., samples of kidney and spleen from five mice in the treatment group infected with three *Treponema* spp. were collected for bacterial culture.

### 2.4. Bacterial Culture

Collected samples were placed in an Eppendorf tube and transported to an anaerobic cabinet. Thereafter, 1 mL of pre-reduced NOS media (supplemented with 10% bovine fetal serum and 10% rabbit serum) was added and incubated at 37 °C. Bacterial growth was assessed using dark-field microscopy, based on *Treponema* morphological characteristics (thin corkscrew-shaped bacteria, 0.1–0.4 μm wide and 4–15 μm long). If no growth was observed after 2 weeks, the culture was considered negative.

### 2.5. Treponema spp. qPCR

A piece of abscess stored at −20 °C was thawed and weighed and DNA extracted using a commercial kit (DNeasy Blood and Tissue Extraction Kit, Qiagen, Hilden, Germany) used in accordance with the manufacturer’s recommendations for Gram-negative bacteria. The recovered DNA was eluted into nuclease-free ultrapure water and its concentration (A_260/280_) was measured with a NanoDrop Spectrophotometer (NanoDrop Technologies, Wilmington, DE, USA).

A species-specific qPCR assay [14] was used for in-tissue *Treponema* quantification. Clones targeting unique genes of *Treponema pedis* (CP004120), *T. phagedenis* (WP_002698807.1) and *T. medium* (WP_016523385.1) were generated and used for quantification. The reaction mixture contained 1× TaqMan^®^ Fast Advanced Master Mix (Applied Biosystems^®^, ThermoFisher, Foster City, CA, USA), 1 μM of each primer and probe, and 2 μL of DNA extract in a final volume of 20 μL. Negative DNA extraction controls, nontemplate DNA and a standards serial dilutions acting as PCR positive controls were included in each PCR assay. Amplification was done in a CFX96 Touch™ Real Time PCR Detection System (Bio-Rad Laboratories, Inc., Hercules, CA, USA) and consisted of 50 °C 2 min, 95 °C 20 s, (95 °C 10 s, 59.6 °C 50 s) × 39, 72 °C 5 min. Results were analyzed using CFX Manager™ Software (Bio-Rad Laboratories, Inc., Hercules, CA, USA). Copy numbers of genes in each sample were transformed to copy number or genomic equivalents per gram of tissue.

### 2.6. Histopathology

After formalin fixation, each skin injection site was bisected longitudinally with a scalpel. Both cut faces were processed for routine paraffin embedding, mounting, haematoxylin and eosin staining and histologic evaluation.

All slides were examined by a veterinary pathologist blinded to the allocation of experimental groups. Lesions were assigned a score, according to six categories: (1) no lesion; (2) minimal lesion, with low numbers of scattered, mixed inflammatory cells, interpreted as a resolving lesion; (3) small, discrete abscess with well organized, concentric laminar architecture, consisting of a dense central core of neutrophils, a middle layer of macrophages, and a peripheral capsule of loose fibrous tissue intermingled with lymphocytes and fewer neutrophils; (4) larger, less well organized but still discrete abscess with a moderate amount of amorphous eosinophilic necrocellular debris intermingled with the central neutrophilic core; (5) larger abscess than category 4, with poorer laminar organization, more abundant central necrocellular debris, and little to no deep fibrous encapsulation; (6) larger abscess than category 5, with even more abundant central necrocellular debris, and extensive inflammatory infiltration into the underlying dorsal skeletal muscle. Photographs of all sections were taken, and the area of each lesion measured using image analysis software (cellSens, Olympus Corporation). A histologic index for each mouse was calculated by multiplying the lesion score (ranging from 1 to 6) by the average lesion area (average of two examined images). Thereafter, data were divided by the highest histologic index obtained and multiplied by 10 (for 1 to 10 scale transformation).

### 2.7. Transcriptional Gene Expression of Innate Immune Factors in Murine Abscesses

Relative messenger gene (mRNA) expression of murine cytokines (CXCL-1, IL-10 and TGF-β) from murine abscesses was quantified by qRT-PCR. Total RNA from each abscess was isolated using Trizol reagent (Ribozol^TM^, VWR International, Mississauga, ON, Canada), according to the manufacturer’s instructions. Complementary DNA (cDNA) was prepared from 1 μg of total RNA using Moloney murine leukemia virus reverse transcriptase (Quanta Biosciences, qScripts cDNA synthesis kit, Qiagen, Mississauga, ON, Canada). The quality and quantity of resulting RNA and cDNA were determined using a NanoVue Spectrophotometer (GE Healthcare, Baie D’Urfe, QC, Canada). Absence of contaminating genomic DNA from RNA preparations was verified using a minus-reverse transcriptase control (i.e., sample with all RT-PCR reagents except reverse transcriptase). Then, qRT-PCR was performed using a CFX-96 real time PCR system (Bio-Rad Laboratories, Inc., Hercules, CA, USA). Each reaction mixture contained 100 ng of cDNA, SYBR Green Real-Time PCR Master Mixes (Thermo Fisher Scientific) and 0.5 μM of each specific primer, in a final volume of 10 μL. Primers for GAPDH (PPM02946E), CXCL-1 (PPM03058C), IL-10 (PPM03017C) and TGF-β (PPM02991B) were used. The PCR reaction consisted of 95 °C 5 min, (95 °C 5 s, 60 °C 10 s) × 40. Two housekeeping genes, GAPDH and β-actin, were initially tested and GAPDH was selected for data normalization based on the transcription stability. Negative controls for cDNA synthesis and PCR procedures were consistently included. Values of target mRNA were corrected relative to the housekeeping gene coding GAPDH. Data were analysed using the 2^−ΔΔ*C*T^ method and results reported as mean fold change of the target transcription levels in all groups versus mice infected with *T. phagedenis* (control group).

### 2.8. Statistical Analyses

Data normality was assessed using a Shapiro-Wilk test. Normally distributed data (abscess size, histology index and relative mRNA fold changes for studied gene expression) were analyzed by one-way analysis of variance (ANOVA), followed by Tukey’s post hoc test to identify differences. Non-normally distributed data (*Treponema* spp. genomic equivalents) were log-transformed to achieve normality. Figures were generated using GraphPad Prism 7 (GraphPad Software Inc., La Jolla, San Diego, CA, USA). For all analyses, *p* < 0.05 was significant.

## 3. Results

### 3.1. DD-Associated Treponema spp. Induce Abscesses of Variable Sizes

**Figure 2:**
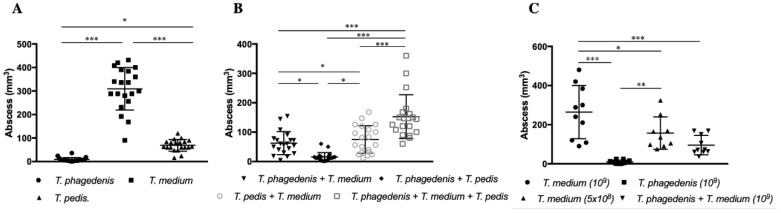
Skin abscess size after subcutaneous inoculation with single or multiple *Treponema* spp. (**A**) Lesion size (mm^3^) in mice infected with single *Treponema* spp. (**B**) Lesion size in mice infected with *Treponema* spp. combinations. (**C**) Skin lesion size in Experiment 2 carried out with single and *Treponema* spp. pair combination. Graphs representing mean and the error bars indicating the standard deviations are presented. * *p* < 0.05, ** *p* < 0.01, *** *p* < 0.001.

All three *Treponema* spp. most commonly isolated from DD were able to induce abscess formation in mice after subcutaneous inoculation of 10^9^ *Treponema* (Figure 2A–C and Table S1) in Experiment 1. No mice in either control group (challenged with formalin-killed *Treponema* spp. or inoculated with PBS) developed any skin lesions 7 days post challenge.

*T. phagedenis* induced abscesses smaller than those produced by *T. medium* (*p* < 0.001) or *T. pedis* (*p* = 0.002), whereas *T. medium* induced the largest abscesses, larger (*p* < 0.0001) than those produced by *T. pedis* (Figure 2A, Table S1). When mice were challenged with pairs of *Treponema* spp. (Figure 2B, Table S1), *T. phagedenis + T. pedis* produced smaller abscesses than either *T. phagedenis* + *T. medium* (*p* = 0.016) or *T. pedis* + *T. medium* (*p* = 0.001).

Abscesses resulting from inoculation of one versus two *Treponema* spp. were compared. *T. phagedenis* alone produced smaller abscesses than either *T. phagedenis* + *T. medium* (*p* = 0.02) or *T. pedis + T. medium* (*p* = 0.001). *T. medium* alone produced larger abscesses than any of the *Treponema* pair combinations (*p* < 0.001); *T. phagedenis + T. medium*, *T. phagedenis + T. pedis* or *T. pedis + T. medium. T. pedis* alone produced larger abscesses than *T. phagedenis* + *T. pedis* (*p* = 0.02).

Mice in the group challenged with a mixture of three *Treponema* species (*T. phagedenis* + *T. medium* + *T. pedis*) developed abscesses larger than those from any of the *Treponema* spp. pair combinations (*p* < 0.001) or single *T. phagedenis* and *T. pedis* inoculation (*p* < 0.0001), but smaller than abscesses resulting from *T. medium* inoculation (*p* < 0.0001).

Experiment 2 was done to assess reproducibility and to investigate dose effects on abscess size (Figure 2C, Table S1). Subcutaneous 10^9^ *T. medium* inoculation induced larger abscesses than either a half dose of *T. medium* (5 × 10^8^) (*p* = 0.04) or a combination of *T. phagedenis* (5 × 10^8^) + *T. medium* (5 × 10^8^) (*p* = 0.002). However, there were no differences between *T. phagedenis* (10^9^) and *T. phagedenis* (5 × 10^8^) *+ T. medium* (5 × 10^8^) (*p* = 0.12). None of the replicated experimental groups had differences in abscess sizes between the first and second experiments (*T. phagedenis*, *p* > 0.99; *T. medium* (10^9^), *p* = 0.502; and *T. phagedenis +T. medium, p* = 0.803), (Figure 2A–C and Table S1), confirming the reproducibility of the results.

### 3.2. Viable Treponema spp. Detected by Culture

*T. medium* was the only *Treponema* sp. isolated by culture when mice were challenged with a single *Treponema* sp. (Table 1). Culture was positive for 80% (8/10) of samples, with an average abscess volume of 312 mm^3^ (±77.9). Regarding inoculation with combinations, *Treponema* were isolated from 20% (2/10) of samples from mice inoculated with *T. phagedenis* + *T. medium*, 30% (3/10) of samples from mice inoculated with *T. medium* + *T. pedis* and 50% (5/10) of samples from mice inoculated with all three *Treponema* spp. (*T. medium* + *T. pedis* + *T. phagedenis*). Abscesses from culture positive samples were significantly larger than the abscesses from culture negative samples (*p* = 0.04).

When kidney and spleen samples from mice infected with a combination of three *Treponema* spp. were cultured, *Treponema* spp. were isolated by culture from 40% (2/5) kidney samples, but not from spleens.

### 3.3. Variable Numbers of Treponema spp. Detected

**Figure 3:**
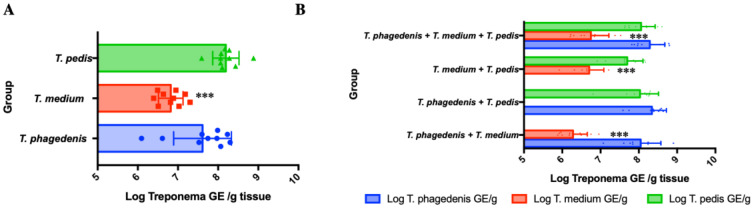
Quantities of Treponema spp. in the abscesses of mice subcutaneously inoculated expressed as log10 of *Treponema* genomic equivalents (GE) per gram of tissue. (**A**) Mice infected with single *Treponema* spp.; and (**B**) mice infected with *Treponema* spp. combinations. Bars represent the mean and error bars indicate standard deviation. *** *p* < 0.001.

*Treponema* spp. presence in the abscesses 7 days post challenge was investigated using qPCR and expressed as logarithmic genomic units (LGE) per g tissue (Figure 3, Table 2). After single *Treponema* species inoculation (Figure 3A, Table 2), there were lower numbers of *T. medium* than *T. phagedenis* (*p* = 0.003) or *T. pedis* (*p* < 0.0001).

The qPCR results from abscesses of mice inoculated with a combination of *Treponema* species were also compared (Figure 3B, Table 2). When mice were inoculated with *T. medium* + *T. phagedenis*, *T. medium* was detected in lower amounts than *T. phagedenis* (*p* < 0.0001). Similarly, in mice inoculated with *T. medium + T. pedis*, *T. medium* was also detected in lower amounts than *T. pedis* (*p* < 0.0001). In mice infected with all three *Treponema* species, there were fewer *T. medium* detected than *T. phagedenis* or *T. pedis* (*p* < 0.001). There were no significant differences between numbers of *T. phagedenis* and *T. pedis* (*p* = 0.094) detected in the *T. phagedenis* + *T. pedis* inoculated mice. Similarly, there were no significant differences between *T. phagedenis* and *T. pedis* quantities in mice challenged with all three species (*p* = 0.266).

A qPCR method was used to identify *Treponema* spp. growth in cultures of kidney samples. One sample yielded *T. phagedenis* whereas the other yielded all three species.

### 3.4. Differences in Treponema spp. Induced Lesion

**Figure 4:**
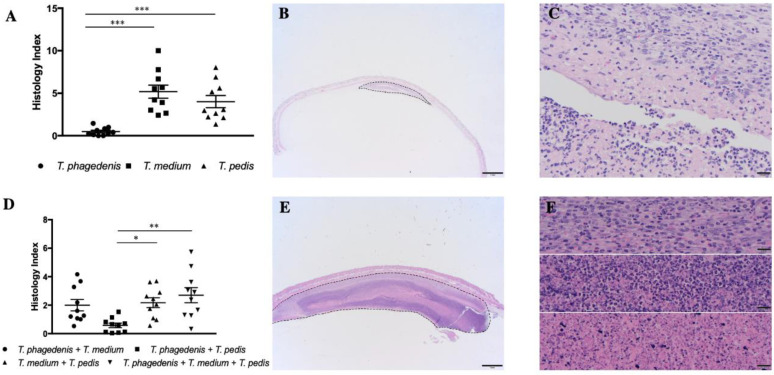
Histopathology of lesions after subcutaneous inoculation with *Treponema* spp. (haematoxylin and eosin staining). Histology index of single *Treponema* spp. infection (**A**) and multiple *Treponema* spp. infection (**D**). *T. phagedenis*-induced subcutaneous abscess, transverse section (area of the abscess outlined 21,355 pixels^2^; scale bar = 1 mm) (**B**) and high magnification view (scale bar = 20 µm) (**C**). The upper part presents the relatively narrow fibrovascular capsule composed of proliferating fibroblasts, among which neutrophils are loosely scattered. Deep to this capsule is a layer of macrophages and a dense layer composed predominantly of neutrophils. *T. medium*-induced subcutaneous abscess, transverse section (area of the abscess outlined 366,519 pixels^2^; scale bar = 1 mm) (**E**) and high magnification view (**F**). The upper image shows the thick fibrovascular capsule that surrounds the abscess, composed primarily of proliferating fibroblasts among which are scattered neutrophils. The middle panel presents the dense layer of neutrophils and cellular debris at the periphery of the abscess. The lower panel presents the amorphous necrocellular debris at the center of the abscess (scale bar = 20 µm). * *p* < 0.05, ** *p* < 0.01, *** *p* < 0.001.

Hematoxylin and eosin-stained tissue sections of all injection sites were evaluated and scored using a scale (histology index) based on size, inflammatory cell infiltration and lesion architecture (Figure 4). In mice inoculated with PBS or formalin-killed *Treponema* spp., neither gross nor microscopic skin lesions were present 7 days post inoculation.

In mice inoculated with a single *Treponema* spp., *T. phagedenis* produced the lowest histology index values when compared to *T. medium* (*p* < 0.0001) and *T. pedis* (*p* < 0.001). However, there were no differences between *T. medium* and *T. pedis* (*p* = 0.38) (Figure 4A).

Inoculation with multiple *Treponema* spp. resulted in a lower histology index for *T. phagedenis + T. pedis* than for *T. medium* + *T. pedis* challenged mice (*p* = 0.027). There were no significant differences among the histology indices of mice infected with *T. phagedenis + T. medium and T. pedis + T. medium* (*p* = 0.99). The group inoculated with all three *Treponema* spp. (*T. phagedenis + T. medium + T. pedis)* had a higher histology index than those challenged with *T. phagedenis + T. pedis* (*p* = 0.002) (Figure 4D).

### 3.5. Treponema Induced Differential Expression of Innate Immune Factors

**Figure 5:**
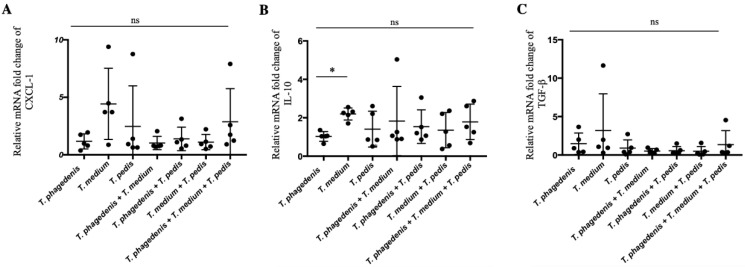
Transcriptional gene expression of Cxcl-1 (**A**), IL-10 (**B**) and TGF-β (**C**) abscess lesions of mice challenged by *Treponema* single and multiple combination of species. Means and standard deviation are presented. Analyzed by ANOVA using Tukey’s post hoc test. * *p* < 0.05.

Relative fold changes in CXCL-1, IL-10 and TGF-β mRNA were studied in skin lesions using the single *T. phagedenis* inoculation group as a control (Figure 5). Chemoattractant CXCL-1 and regulatory TGF-β cytokine transcription expression were not different among any of the challenged groups (Figure 4A–C) (*p* > 0.59). Increased expression of anti-inflammatory IL-10 was detected in abscesses of *T. medium*-challenged mice in comparison to *T. phagedenis*-inoculated mice (*p* = 0.02). Mice inoculated with all three *Treponema* spp. did not have differences in IL-10 gene expression (*p* > 0.99).

## 4. Discussion

In this study, subcutaneous abscess formation was confirmed in mice 7 days after inoculation with the most common *Treponema* spp. isolated from active bovine DD lesions. A murine subcutaneous abscess model was used to evaluate oral *Treponema* spp. [26,30] or determine pathogenicity of *T. phagedenis* isolated from DD lesions [32]. However, to the best of our knowledge, this was the first study to evaluate *T. medium* and *T. pedis* pathogenicity in mice and to compare pathogenicity of single versus multiple *Treponema* spp.

Although *Treponema* spp. derived from DD and other sources can be genetically very similar [37], differential pathogenicity has been associated with adaptation to various environments (oral cavity, genitalia etc.). *Treponema phagedenis* Kazan 8 (originally identified as *T. pallidum,* ATCC 27087) is a nonpathogenic member of the resident human genital microflora [38] and in mice induces smaller abscess formation than DD-isolated *T. phagedenis* [32]. As *Treponema phagedenis* is one of the most commonly isolated *Treponema* species from DD lesions [39,40,41], it could be expected to have a central role in lesion development. However, in the present study, *T. phagedenis,* when inoculated alone, induced smaller lesions than other treponemes, yielding abscess sizes similar to those previously reported at the same infectious dose [32]. Interestingly, experimental infection with a pure culture of *T. phagedenis* in a bovine model has had limited success [34], suggesting that this *Treponema* species alone will likely have a limited or perhaps no role in DD pathogenesis.

In both in vivo experiments, inoculation with *T. medium* alone produced larger abscesses than inoculation with either *T. phagedenis* or *T. pedis* alone. It also produced larger abscesses alone than when inoculated in combination with either *T. phagedenis* or *T. pedis*. Despite no significant difference between *T. medium* and *T. pedis* for the histology index, there were significant differences between *T. medium* and *T. phagedenis*. In previous studies using fluorescence in situ hybridization (FISH), various *Treponema* species occupied different tissue layers [11,39]. Whereas *T. phagedenis* is present in all skin layers, *T. medium* is mostly detected deeper, at the interface between damaged and healthy epidermis [42]. Furthermore, greater numbers of *T. medium* were present deeper in active lesions, in contrast to *T. phagedenis* [43]. *Treponema* spp. localization in skin lesions could be related to oxygen sensitivity, a poorly studied aspect in DD-associated *Treponema* spp. In a previous study, whereas *T. phagedenis* and *T. pedis* were recovered from the gloves of hoof trimmers after 2 or 3 days, *T. medium* was only recoverable for 1 day [44], suggesting it may be more sensitive to oxygen, consistent with localization and survival of *T. medium* deeper within skin lesions. *Treponema medium* is one of the more prevalent *Treponema* spp. in human endodontic abscesses [45], and noncultivable *Treponema* that are closely related to *T. medium* are more prevalent in bovine ulcerative mammary dermatitis [46], indicating that *T. medium* could be more pathogenic than other *Treponema* spp.

In our present study, no synergistic effects were detected when mice were inoculated with mixed *Treponema* spp. Supporting this finding, a mixture of DD *T. phagedenis* isolates did not produce synergistic effects [32]. Species of Treponema constitute the main pathogens linked to DD. However, some studies have also proposed *Dichelobacter *nodosus**, *Fusobacterium necrophorum* and *Porphyromonas levii* as potentially important pathogens involved in this complex disease [39]. Therefore, potential synergistic effects between these bacteria should be further investigated to elucidate their role in DD pathogenesis.

In our experiments, number of *Treponema* isolated from bacterial culture was positively associated with abscess size. Anaerobic culture of treponemes was more frequent in larger abscesses, suggesting that *T. medium,* which produces bigger abscess, remains viable in tissue longer than other *Treponema* spp. We reported that culture of DD-associated *Treponema* is not successful when only a low number of spirochetes are present [36], suggesting that small abscess may also harbour live spirochetes, but which are not able to be cultured. Surprisingly, in our experiments, *Treponema* spp. were cultured from kidney samples, demonstrating that systemic infections after subcutaneous infection may occur in mice. Species identification by qPCR of *Treponema* spp. cultured from kidneys (*T. phagedenis*, *T. medium* and *T. pedis*) indicated that all inoculated species have the capacity for systemic dissemination. *Treponema* spp. dissemination in mice was confirmed when *T. denticola* DNA was detected in the spleen, heart and brain after endodontic infection [47]. Further evidence of *Treponema* dissemination in mice comes from the detection of *T. denticola* clusters in aortic tissue by FISH, confirming a causal link between active oral *T. denticola* infection and atheroma or periodontal disease [48]. Unfortunately, kidney samples were not included in any of those studies. This indicates the need for further studies to determine which *Treponema* species can invade the kidney, as seen with other spirochetes such as *T. pallidum* [49], *Borrelia burgdorferi* [50] and *Leptospira* [51]. The presence of DD-associated *Treponema* spp. in the kidneys and urine of infected animals needs to be further investigated, as infection reservoirs and transmission routes of DD remain unclear.

In the present study, qPCR detected fewer *T. medium* in all groups (single or multiple species inoculation). This was surprising, as *T. medium* produced the largest abscesses and it was the only single *Treponema* spp. recovered by culture. However, DD-associated *Treponema* spp. develop cystic or resistant forms during in vitro culture [52]. There is lack of knowledge on whether some *Treponema* species have a higher ability to produce cystic forms or if they are induced during in vivo infection as described for other spirochetes [53]. In that regard, the potential presence of more cystic forms of *T. medium*, associated with a lower DNA extraction efficiency (resistant forms), could explain the obtained results.

There was higher transcriptional expression of IL-10 for *T. medium* versus *T. phagedenis*. It is noteworthy that IL-10 plays a role in suppressing inflammatory reactions, including in skin disorders [54] and downregulation of IL-10 occurs in active DD [55]. Chemoattractant CXCL-1 and regulatory TGF-β were unaffected, indicating responses of those factors could occur at posttranscriptional level or at later points.

Our study showed that there was varying pathogenicity among DD-associated *Treponema* spp. in a murine subcutaneous abscess model, suggesting that some *Treponema* species, although present in lower abundance, may have more impact on the development of DD lesions than those with larger numbers. In addition, *Treponema* spp. systemic infection was confirmed. Further studies in cattle and including more isolates are required to validate our findings.

## Figures and Tables

**Table 1:** *Treponema* spp. isolated in culture from abscesses of inoculated mice.

Group	Culture (%) ^2^	Average Size ^1^ (SD)	
		Culture Positive	Culture Negative
*T. phagedenis*	0	-	5.3 (1.89)
*T. medium*	80	311.9 (77.92)	228.0 (84.85)
*T. pedis*	0	-	72.9 (17.62)
*T. phagedenis + T. medium*	20	149.0 (7.07)	40.6 (27.86)
*T. phagedenis + T. pedis*	0	-	22.2 (18.12)
*T. medium + T. pedis*	30	124.0 (3.46)	57.0 (39.32)
*T. phagedenis + T. medium + T. pedis*	50	173.8 (48.62)	120.4 (26.84)

^1^ Size (mm^3^). ^2^ Percentage of positive *Treponema* spp. cultured samples.

**Table 2:** Mean (SD) *Treponema* spp. quantities detected by qPCR in the abscesses of inoculated mice. Quantities expressed as logarithmic genomic equivalents (LGE) per gram of tissue.

Group	*T. phagedenis* LGE/g Tissue (SD)	*T. medium* LGE/g Tissue (SD)	*T. pedis* LGE/g Tissue (SD)
*T. phagedenis*	7.6 (0.72)	-	-
*T. medium*	-	6.8 (0.30)	-
*T. pedis*	-	-	8.2 (0.33)
*T. phagedenis + T. medium*	8.0 (0.53)	6.3 (0.37)	-
*T. phagedenis + T. pedis*	8.3 (0.38)	-	8.0 (0.48)
*T. medium + T. pedis*	-	6.7 (0.38)	7.7 (0.4)
*T. phagedenis + T. medium + T. pedis*	8.3 (0.39)	6.7 (0.47)	8.1 (0.37)

## Supplementary Materials

The following are available online at https://www.mdpi.com/2076-2607/8/10/1507/s1, Table S1: Mean and SD abscess size (mm^3^) after subcutaneous inoculation of mice with 10^9^ (Experiment 1) or 10^9^ or 5 × 10^8^ (Experiment 2) *Treponema* spp.

## References

[1] Holzhauer M., Bartels C.J.M., Döpfer D., van Schaik G. (2008). Clinical course of digital dermatitis lesions in an endemically infected herd without preventive herd strategies. Vet. J..

[2] Berry S.L. (2001). Diseases of the digital soft tissues. Vet. Clin. N. Am. Food Anim. Pract..

[3] Losinger W.C. (2006). Economic impacts of reduced milk production associated with papillomatous digital dermatitis in dairy cows in the USA. J. Dairy Res..

[4] Cha E., Hertl J.A., Bar D., Gröhn Y.T. (2010). The cost of different types of lameness in dairy cows calculated by dynamic programming. Prev. Vet. Med..

[5] Bruijnis M.R.N., Beerda B., Hogeveen H., Stassen E.N. (2012). Assessing the welfare impact of foot disorders in dairy cattle by a modeling approach. Animal.

[6] Gotoh Y., Chiba K., Sekiyama Y., Okada K., Hayashi T., Misawa N. (2020). 16S rRNA-based amplicon analysis of changes in the bacterial population in the lesions of papillomatous digital dermatitis in dairy cattle after topical treatment with allyl isothiocyanate. Microbiol. Immunol..

[7] Nielsen M.W., Strube M.L., Isbrand A., Al-Medrasi W.D.H.M., Boye M., Jensen T.K., Klitgaard K. (2016). Potential bacterial core species associated with digital dermatitis in cattle herds identified by molecular profiling of interdigital skin samples. Vet. Microbiol..

[8] Krull A.C., Shearer J.K., Gorden P.J., Cooper V.L., Phillips G.J., Plummer P.J. (2014). Deep sequencing analysis reveals temporal microbiota changes associated with development of bovine digital dermatitis. Infect. Immun..

[9] Evans N.J., Murray R.D., Carter S.D. (2016). Bovine digital dermatitis: Current concepts from laboratory to farm. Vet. J..

[10] Evans N.J., Brown J.M., Demirkan I., Singh P., Getty B., Timofte D., Vink W.D., Murray R.D., Blowey R.W., Birtles R.J. (2009). Association of unique, isolated treponemes with bovine digital dermatitis lesions. J. Clin. Microbiol..

[11] Klitgaard K., Boye M., Capion N., Jensen T.K. (2008). Evidence of multiple Treponema phylotypes involved in bovine digital dermatitis as shown by 16S rRNA gene analysis and fluorescence in situ hybridization. J. Clin. Microbiol..

[12] Nordhoff M., Moter A., Schrank K., Wieler L.H. (2008). High prevalence of treponemes in bovine digital dermatitis-A molecular epidemiology. Vet. Microbiol..

[13] Orsel K., Plummer P., Shearer J., De Buck J., Carter S.D., Guatteo R., Barkema H.W. (2018). Missing pieces of the puzzle to effectively control digital dermatitis. Transbound. Emerg. Dis..

[14] Beninger C., Naqvi S.A., Naushad S., Orsel K., Luby C., Derakhshani H., Khafipour E., De Buck J. (2018). Associations between digital dermatitis lesion grades in dairy cattle and the quantities of four Treponema species. Vet. Res..

[15] Demirkan I., Erdoğan M., Demirkan A.Ç., Bozkurt F., Altındiş M., Navruz F.Z., Köse Z. (2018). Isolation and identification of *Treponema pedis* and *Treponema phagedenis*-like organisms from bovine digital dermatitis lesions found in dairy cattle in Turkey. J. Dairy Sci..

[16] Sullivan L.E., Clegg S.R., Angell J.W., Newbrook K., Blowey R.W., Carter S.D., Bell J., Duncan J.S., Grove-White D.H., Murray R.D. (2015). High-level association of bovine digital dermatitis *Treponema* spp. with contagious ovine digital dermatitis lesions and presence of *Fusobacterium necrophorum* and *Dichelobacter nodosus*. J. Clin. Microbiol..

[17] Han S., Mansfield K.G., Bradway D.S., Besser T.E., Read D.H., Haldorson G.J., Alt D.P., Wilson-Welder J.H. (2019). Treponeme-associated hoof disease of free-ranging elk (*Cervus elaphus*) in Southwestern Washington State, USA. Vet. Pathol..

[18] Clegg S.R., Carter S.D., Stewart J.P., Amin D.M., Blowey R.W., Evans N.J. (2016). Bovine ischaemic teat necrosis: A further potential role for digital dermatitis treponemes. Vet. Rec..

[19] Clegg S.R., Sullivan L.E., Bell J., Blowey R.W., Carter S.D., Evans N.J. (2016). Detection and isolation of digital dermatitis treponemes from skin and tail lesions in pigs. Res. Vet. Sci..

[20] Lamont R.J., Hajishengallis G. (2015). Polymicrobial synergy and dysbiosis in inflammatory disease. Trends Mol. Med..

[21] Tan K.H., Seers C.A., Dashper S.G., Mitchell H.L., Pyke J.S., Meuric V., Slakeski N., Cleal S.M., Chambers J.L., McConville M.J. (2014). *Porphyromonas gingivalis* and *Treponema denticola* exhibit metabolic symbioses. PLoS Pathog..

[22] Grenier D. (1992). Nutritional interactions between two suspected periodontopathogens, *Treponema denticola* and *Porphyromonas gingivalis*. Infect. Immun..

[23] Zhu Y., Dashper S.G., Chen Y.Y., Crawford S., Slakeski N., Reynolds E.C. (2013). *Porphyromonas gingivalis* and *Treponema denticola* synergistic polymicrobial biofilm development. PLoS ONE.

[24] Yamada M., Ikegami A., Kuramitsu H.K. (2005). Synergistic biofilm formation by *Treponema denticola* and *Porphyromonas gingivalis*. FEMS Microbiol. Lett..

[25] Orth R.K.-H., O’Brien-Simpson N.M., Dashper S.G., Reynolds E.C. (2011). Synergistic virulence of *Porphyromonas gingivalis* and *Treponema denticola* in a murine periodontitis model. Mol. Oral Microbiol..

[26] Kesavalu L., Holt S.C., Ebersole J.L. (1998). Virulence of a polymicrobic complex, *Treponema denticola* and *Porphyromonas gingivalis*, in a murine model. Oral Microbiol. Immunol..

[27] Bodet C., Chandad F., Grenier D. (2007). Potentiel pathogénique de *Porphyromonas gingivalis*, *Treponema denticola* et *Tannerella forsythia*, le complexe bactérien rouge associé à la parodontite. Pathol. Biol..

[28] Hajishengallis G., Lamont R.J. (2012). Beyond the red complex and into more complexity: The polymicrobial synergy and dysbiosis (PSD) model of periodontal disease etiology. Mol. Oral Microbiol..

[29] Abe T., Hajishengallis G. (2013). Optimization of the ligature-induced periodontitis model in mice. J. Immunol. Methods.

[30] Kesavalu L., Walker S.G., Holt S.C., Crawley R.R., Ebersole J.L. (1997). Virulence characteristics of oral treponemes in a murine model. Infect. Immun..

[31] Washizu M., Ishihara K., Honma K., Okuda K. (2003). Effects of a mixed infection with *Porphyromonas gingivalis* and *Treponema denticola* on abscess formation and immune responses in mice. Bull. Tokyo Dent. Coll..

[32] Elliott M.K., Alt D.P., Zuerner R.L. (2007). Lesion formation and antibody response induced by papillomatous digital dermatitis-associated spirochetes in a murine abscess model. Infect. Immun..

[33] Gomez A., Cook N.B., Bernardoni N.D., Rieman J., Dusick A.F., Hartshorn R., Socha M.T., Read D.H., Döpfer D. (2012). An experimental infection model to induce digital dermatitis infection in cattle. J. Dairy Sci..

[34] Krull A.C., Cooper V.L., Coatney J.W., Shearer J.K., Gorden P.J., Plummer P.J. (2016). A Highly effective protocol for the rapid and consistent induction of digital dermatitis in holstein calves. PLoS ONE.

[35] Wilson-Welder J.H., Nally J.E., Alt D.P., Palmer M.V., Coatney J., Plummer P. (2018). Experimental transmission of bovine digital dermatitis to sheep: Development of an infection model. Vet. Pathol..

[36] Arrazuria R., Caddey B., Cobo E.R., Barkema H.W., DeBuck J. (2020). Digital dermatitis *Treponema* spp. mixed community responds differently to culture media and serum supplementation. Anaerobe.

[37] Clegg S.R., Carter S.D., Birtles R.J., Brown J.M., Anthony Hart C., Evans N.J. (2016). Multilocus sequence typing of pathogenic treponemes isolated from cloven-hoofed animals and comparison to treponemes isolated from humans. Appl. Environ. Microbiol..

[38] Willcox R.R., Guthe T. (1966). *Treponema pallidum*. A bibliographical review of the morphology, culture and survival of *T. pallidum* and associated organisms: Introduction. Bull. World Health Organ..

[39] Moreira T.F., Facury Filho E.J., Carvalho A.U., Strube M.L., Nielsen M.W., Klitgaard K., Jensen T.K. (2018). Pathology and bacteria related to digital dermatitis in dairy cattle in all year round grazing system in Brazil. PLoS ONE.

[40] Pringle M., Bergsten C., Fernström L.-L., Höök H., Johansson K.-E. (2008). Isolation and characterization of *Treponema phagedenis*-like spirochetes from digital dermatitis lesions in Swedish dairy cattle. Acta Vet. Scand..

[41] Wilson-Welder J.H., Elliott M.K., Zuerner R.L., Bayles D.O., Alt D.P., Stanton T.B. (2013). Biochemical and molecular characterization of *Treponema phagedenis*-like spirochetes isolated from a bovine digital dermatitis lesion. BMC Microbiol..

[42] Klitgaard K., Bretó A.F., Boye M., Jensen T.K. (2013). Targeting the treponemal microbiome of digital dermatitis infections by high-resolution phylogenetic analyses and comparison with fluorescent in situ hybridization. J. Clin. Microbiol..

[43] Zinicola M., Lima F., Lima S., Machado V., Gomez M., Döpfer D., Guard C., Bicalho R. (2015). Altered microbiomes in bovine digital dermatitis lesions, and the gut as a pathogen reservoir. PLoS ONE.

[44] Angell J.W., Clegg S.R., Grove-White D.H., Blowey R.W., Carter S.D., Duncan J.S., Evans N.J. (2017). Survival of contagious ovine digital dermatitis (CODD)-Associated treponemes on disposable gloves after handling CODD-Affected feet. Vet. Rec..

[45] Siqueira J.F., Rocas I.N. (2004). Treponema species associated with abscesses of endodontic origin. Oral Microbiol. Immunol..

[46] Stamm L.V., Walker R.L., Read D.H. (2009). Genetic diversity of bovine ulcerative mammary dermatitis-associated Treponema. Vet. Microbiol..

[47] Foschi F., Izard J., Sasaki H., Sambri V., Prati C., Müller R., Stashenko P. (2006). *Treponema denticola* in disseminating endodontic infections. J. Dent. Res..

[48] Chukkapalli S.S., Rivera M.F., Velsko I.M., Lee J.Y., Chen H., Zheng D., Bhattacharyya I., Gangula P.R., Lucas A.R., Kesavalu L. (2014). Invasion of oral and aortic tissues by oral spirochete *Treponema denticola* in ApoE-/- mice causally links periodontal disease and atherosclerosis. Infect. Immun..

[49] Salazar J.C., Rathi A., Michael N.L., Radolf J.D., Jagodzinski L.L. (2007). Assessment of the kinetics of *Treponema pallidum* dissemination into blood and tissues in experimental syphilis by real-time quantitative PCR. Infect. Immun..

[50] Schwarzova K., Ciznar I., Svihrova V., Hudeckova H. (2019). Initial attachment of *Borrelia burgdorferi* spirochetes to Vero cells. Bratislava Med. J..

[51] Chiu S.-H., Pan M.-J. (2019). Detection and Treatment of Leptospirosis Kidney Disease. Leptospirosis and the Kidney.

[52] Döpfer D., Anklam K., Mikheil D., Ladell P. (2012). Growth curves and morphology of three Treponema subtypes isolated from digital dermatitis in cattle. Vet. J..

[53] Miklossy J., Kasas S., Zurn A.D., McCall S., Yu S., McGeer P.L. (2008). Persisting atypical and cystic forms of *Borrelia burgdorferi* and local inflammation in Lyme neuroborreliosis. J. Neuroinflammation.

[54] Saraiva M., O’Garra A. (2010). The regulation of IL-10 production by immune cells. Nat. Rev. Immunol..

[55] Watts K.M., Fodor C., Beninger C., Lahiri P., Arrazuria R., De Buck J., Knight C.G., Orsel K., Barkema H.W., Cobo E.R. (2018). A differential innate immune response in active and chronic stages of bovine infectious digital dermatitis. Front. Microbiol..

