# Supplementary material for: Treponema spp. Isolated from Bovine Digital Dermatitis Display Different Pathogenicity in a Murine Abscess Model
Source: Microorganisms. 2020 Sep 30;8(10):1507. doi: 10.3390/microorganisms8101507 (PMC7600977; doi:10.3390/microorganisms8101507)
Supplement: Supplementary file 1 [file microorganisms-08-01507-s001.pdf]

Supplementary Table S1. Mean and SD abscess size (mm^3) after subcutaneous inoculation of mice with 10^9 (Experiment 1) or 10^9 or $5x10^8$ (Experiment 2) *Treponema* spp.

**Experiment 1**	**Mean (mm^3)**	**SD**
T. phagedenis	8.35	8.6
T. medium	309.4	90.4
T. pedis	68.9	24.9
T. phagedenis + T. med	62.5	39.3
T. phagedenis + T. pedis	15.5	15
T. medium+ T. pedis	75.3	46.7
T. phagedenis + T. medium + T. pedis	152.6	74.5

Experiment 2		
T. medium(1x10^9)	264	135.9
T. medium(5x10^8)	157	82.9
T. phagedenis (1x10^9)	9.7	9.2
T. phagedenis + T. medium (1x10^9)	95.2	49.3
